# Minimally Invasive Revision Surgery Combining Endoscopic Double-Row Reinsertion and Flexor Hallucis Longus Transfer for Insertional Achilles Tendon Re-Rupture in a Semi-Professional Rugby Player: A Case Report

**DOI:** 10.3390/jcm15197725

**Published:** 2026-10-07

**Authors:** André Santos-Moreira, Marine Riou, Pierre Wagner, Ronny Lopes

**Affiliations:** 1 Serviço de Ortopedia e Traumatologia, Unidade Local de Saúde de Braga, 4710-243 Braga, Portugal; 2 Foot and Ankle Surgery, Centre Orthopédique Santy, FIFA Medical Centre of Excellence, Groupe Ramsay-Générale de Santé, Hôpital Privé Jean Mermoz, 69008 Lyon, France; marine.riou94@gmail.com (M.R.); wagner.pierre@hotmail.fr (P.W.); docteurronnylopes@gmail.com (R.L.)

**Keywords:** insertional Achilles tendon rupture, revision surgery, endoscopic SpeedBridge, flexor hallucis longus transfer, return to sport, case report

## Abstract

**Background/Objectives**: Acute insertional Achilles tendon rupture (A-IATR) is a rare, often season-ending injury in athletes. Revision after a failed repair is particularly challenging, and return to sport (RTS) after such surgery is rarely documented. We report one such case. **Methods**: A 32-year-old semi-professional rugby prop forward sustained an insertional re-rupture eighteen months after open transosseous reinsertion without suture anchors. MRI showed complete detachment with 15 mm of retraction and no residual tendon on the footprint. A single-stage procedure combining endoscopic double-row (SpeedBridge™) reinsertion and flexor hallucis longus (FHL) transfer was performed, followed by six weeks of non-weight-bearing cast immobilization and criteria-based rehabilitation, with Ankle-GO™-guided return to play. **Results**: At three months, MRI showed tendon continuity without re-rupture. Return to play was authorized at 8.2 months (Ankle-GO™ 16/25); the patient returned to official competition at 9.4 months and was still competing approximately 17 months after surgery. At 15 months, the Achilles Tendon Total Rupture Score was 84 and the AOFAS score 90. No complication occurred. **Conclusions**: To our knowledge, this is the first report of RTS after this combined endoscopic revision technique. This single case suggests that this approach may be a feasible revision option in high-demand athletes; comparative studies are needed.

## 1. Introduction

The Achilles tendon plays an important role in sports performance. In high-impact sports, such as rugby, repetitive loading, rapid acceleration/deceleration, and frequent changes of direction impose substantial mechanical demand on the Achilles-calcaneal complex [1].

Acute insertional Achilles tendon rupture (A-IATR) is a rare sports-related injury characterized by avulsion of the distal Achilles tendon sleeve from its insertion on the calcaneal tuberosity [2]. In professional athletes, A-IATR often represents a season-ending injury that requires surgical treatment and an extended period of rehabilitation before returning to sport.

Operative management differs from classic midsubstance Achilles tendon ruptures [2], as it relies on tendon-to-bone healing rather than tendon-to-tendon healing, which has been shown to be associated with less optimal outcomes [3]. This may represent a surgical challenge, as there is no remaining tendon on the calcaneus for repair to the free proximal end, and the avulsed tendon contains insufficient bone to allow internal fixation to the calcaneal tuberosity [4].

Open reinsertion using transosseous sutures or suture anchors has traditionally been considered the standard surgical treatment for A-IATR. However, open reinsertion may be associated with wound-healing complications and prolonged recovery, which are critical in professional athletes. The endoscopic calcaneal SpeedBridge^TM^ (Arthrex, Naples, FL, USA) enables knotless double-row reinsertion through small portals [5,6]. Although evidence specifically addressing A-IATR remains limited, this minimally invasive approach has been associated with a lower complication rate [7] and faster early recovery than open surgery [8] in the treatment of chronic insertional Achilles tendinopathy. These potential advantages may be particularly relevant in high-demand athletes.

In addition, flexor hallucis longus (FHL) transfer may provide biological and mechanical augmentation of the repair, which may be important in revision surgery, where prior open reinsertion can compromise tendon quality and the local soft-tissue envelope.

Despite increasing evidence supporting minimally invasive surgery for Achilles tendon ruptures, return to competitive rugby after revision surgery for sleeve avulsions is rarely reported. Furthermore, the evidence on endoscopic Achilles reinsertion combined with FHL augmentation remains sparse, as most published studies have focused on primary and chronic cases [9,10,11] rather than revision surgery. We describe a single-stage minimally invasive revision procedure combining endoscopic double-row (SpeedBridge^TM^) (Arthrex, Naples, FL, USA) Achilles reinsertion with FHL transfer in a semi-professional rugby player, who returned to official competition 9.4 months after surgery.

## 2. Materials and Methods

### 2.1. History and Clinical Examination

A 32-year-old semi-professional rugby player (prop forward, French fourth division; height 170 cm, weight 106 kg, body mass index 36.7 kg/m^2^; right lower limb dominant) presented with acute right posterior heel pain following sudden ankle dorsiflexion during an acceleration movement in a match (day 0). He was a non-smoker, took no regular medication, had no comorbidity, had never received a corticosteroid injection or fluoroquinolone treatment, and had no relevant family or psychosocial history. Eighteen months earlier, he had sustained a complete insertional (sleeve-type) avulsion of the right Achilles tendon without bony fragment and without previous symptomatic Achilles tendinopathy. This had been treated by open transosseous reinsertion without suture anchors through a medial parasagittal approach, without associated calcaneoplasty. The postoperative course had been free of wound or infectious complications; he had resumed running at 7 months and returned to competition at the same level at 9 months. Clinical examination revealed a palpable gap on calcaneal tendon insertion, weakness of ankle plantarflexion, an increased resting ankle dorsiflexion, and a positive Thompson test.

### 2.2. Diagnostic Assessment

**Figure 1:**
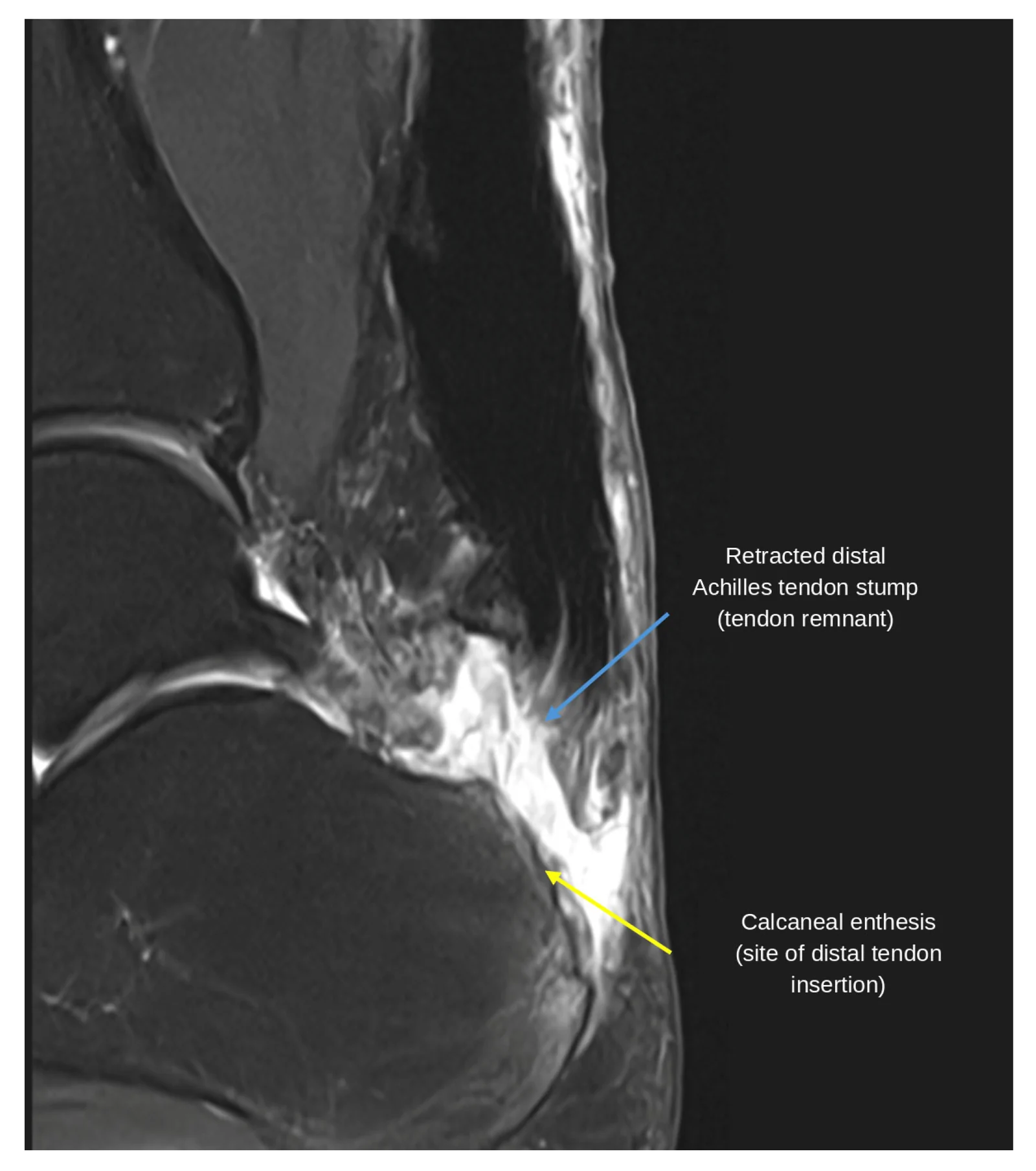
Pre-operative MRI of the right ankle—Sagittal T2-weighted fat-suppressed (T2 Fat-Sat) view (day 3) demonstrating complete recurrent insertional detachment of the Achilles tendon. Blue arrow: retracted distal Achilles tendon stump with diffuse high signal intensity. Yellow arrow: calcaneal enthesis devoid of residual tendon tissue.

Ultrasonography performed on day 1 showed a rupture of the Achilles tendon at its calcaneal insertion. Magnetic resonance imaging (MRI) performed on day 3 confirmed a complete recurrent detachment of the distal Achilles tendon from the calcaneal tuberosity, with approximately 15 mm of fiber retraction (Figure 1). No residual tendon tissue remained attached to the calcaneal enthesis, and the retracted proximal stump showed diffuse high signal intensity, which precluded reliable assessment of its intrinsic quality. The FHL muscle and tendon appeared normal. Given the absence of residual tendon on the footprint, the retraction and the uncertain quality of the stump in a revision setting, isolated revision reinsertion was considered potentially insufficient, and FHL augmentation was planned. Revision surgery was performed on day 9.

### 2.3. Surgical Technique

**Figure 2:**
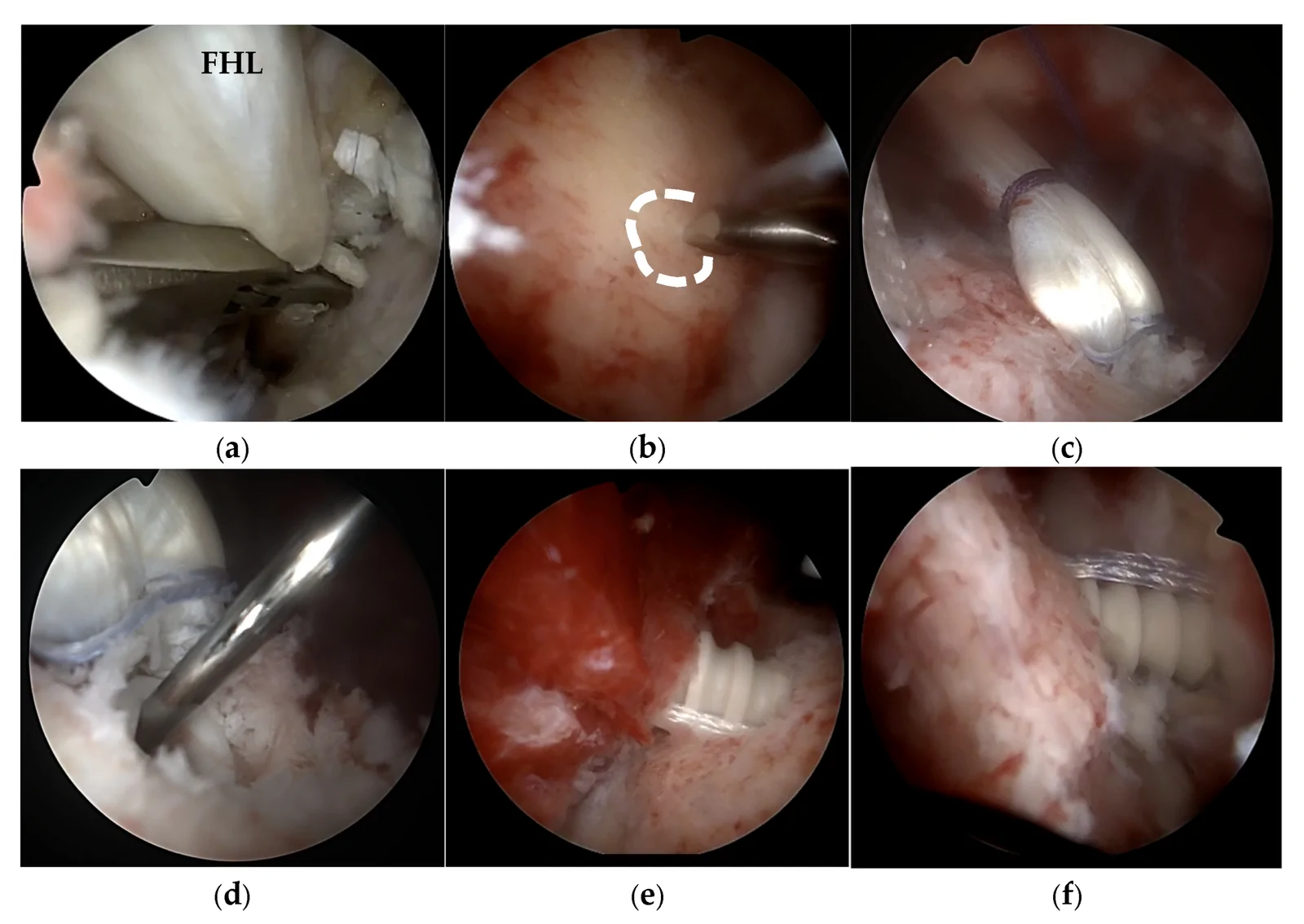
All-endoscopic combined flexor hallucis longus (FHL) transfer with double-row Achilles tendon reinsertion. (**a**) Transection of FHL with a curved-tip arthroscopic scissor in maximum ankle and hallux plantarflexion. (**b**) Visualization of the drill entry point (dashed line) for calcaneal bony tunnel. (**c**) FHL tendon shuttling into the calcaneal tunnel and (**d**) fixation with a 6 × 15 mm Bio-Tenodesis Screw (Arthrex, Naples, FL, USA). (**e**,**f**) Calcaneal SpeedBridge^TM^ (Arthrex, Naples, FL, USA) reinsertion using a knotless double-row repair technique with 4.75 mm SwiveLock^®^ anchors (Arthrex, Naples, FL, USA) and FiberTape^®^ (Arthrex, Naples, FL, USA).

Under epidural anesthesia, with the patient in a prone position and a thigh tourniquet, a single-stage two-part procedure was performed. Posterolateral and posteromedial portals, as described for posterior ankle arthroscopy by Van Dijk [12], allowed release of Kager’s fat pad and creation of a working space with a 3.5 mm shaver. The peroneal tendons and flexor hallucis longus (FHL) tendon were identified as lateral and medial anatomical limits, respectively. First, after its identification, the FHL fibro-osseous tunnel (pulley) was opened endoscopically through the superior portals, and the FHL tendon was completely released of all adhesions to ensure its unrestricted mobility during transfer. To maximize graft length, a traction suture was placed on the proximal portion of the tendon and pulled proximally, and the tendon was transected as distally as possible in the medial retromalleolar groove with a curved-tip arthroscopic scissor (Figure 2a), with the ankle and hallux in maximum plantarflexion. The harvested tendon measured 5 mm in diameter; its length was not measured. Endoscopic calcaneoplasty and debridement allowed to plan and visualize the drill entry point for a complete 6 mm calcaneal bone tunnel, which was drilled through an accessory distal midline portal in a standard fashion (from posterior-superior to inferior-plantar) (Figure 2b). The FHL tendon was subsequently shuttled into the tunnel (Figure 2c) and fixed with a 6 × 15 mm Bio-Tenodesis Screw (Arthrex, Naples, FL, USA) with the ankle in maximum equinus (Figure 2d). The FHL transfer was fixed first because the Achilles tendon is subsequently reinserted onto the tuberosity posterior to the FHL tunnel. Second, the Achilles footprint was visualized through the superior and middle medial and lateral portals, and a burr calcaneoplasty was performed to debride and freshen the footprint. As the index procedure had been a transosseous reinsertion without anchors, no hardware had to be removed. Approximately 5 mm of degenerated distal tendon was debrided, and the retracted stump was reduced to the footprint by traction with the ankle in equinus. Endoscopic calcaneal SpeedBridge^TM^ (Arthrex, Naples, FL, USA) reinsertion was performed [5] using the previously published six-portal knotless double-row technique [6] with four 4.75 mm SwiveLock^®^ anchors (Arthrex, Naples, FL, USA) (Figure 2e,f) and two FiberTape^®^ (Arthrex, Naples, FL, USA): the two proximal-row anchors were inserted first, the tapes were passed transtendinously, and the two distal-row anchors were then inserted and tensioned with the ankle in equinus. No specific dissection of the sural nerve was performed; the portals were placed as described in the published technique. The skin portals were closed with simple sutures.

### 2.4. Postoperative Rehabilitation

**Figure 3:**
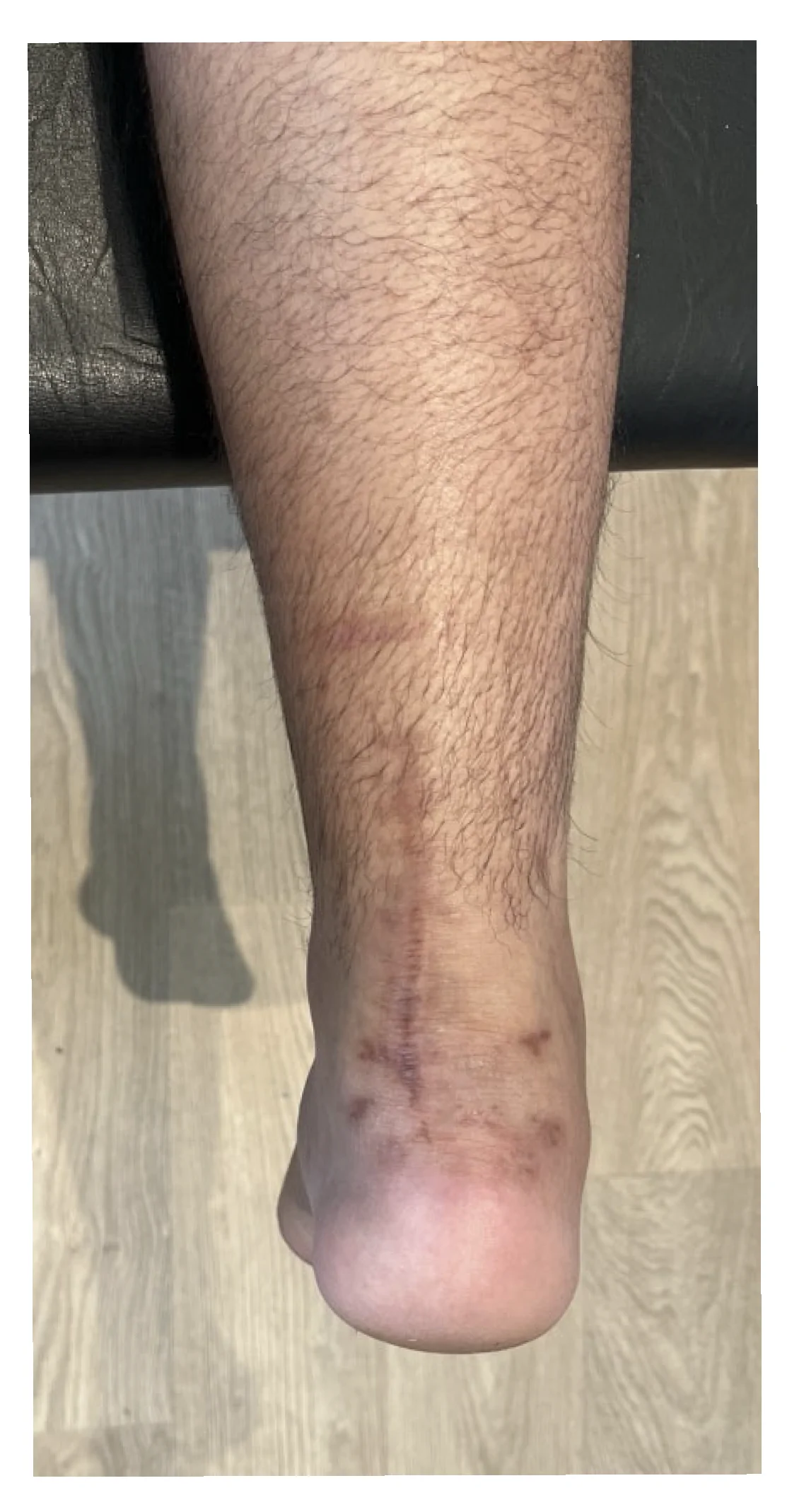
Postoperative scar at 2 months follow-up.

**Figure 4:**
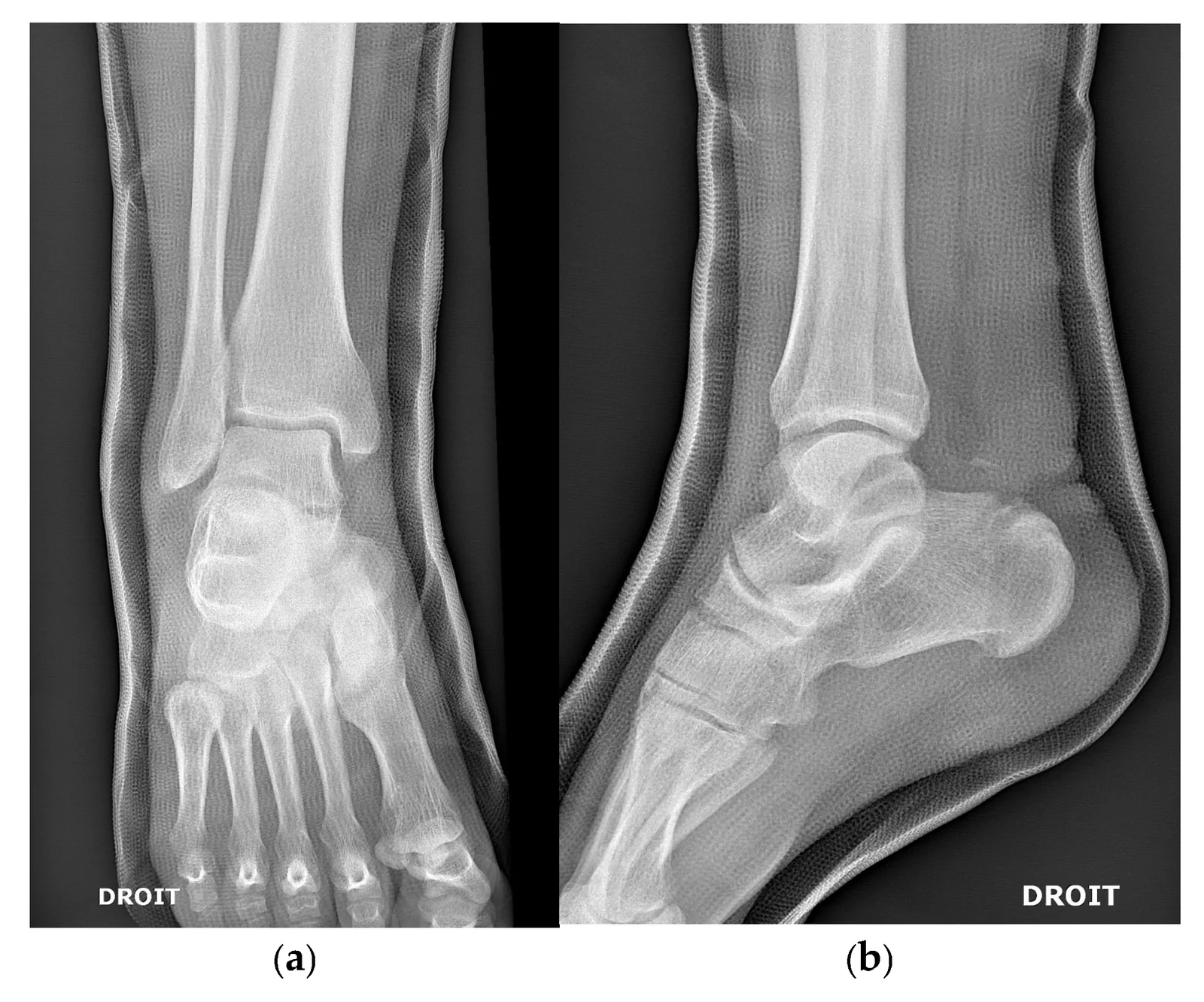
Post-operative radiographs of the right foot and ankle. (**a**) Antero-posterior and (**b**) lateral views showing satisfactory position of the calcaneal anchors.

After surgery, the patient remained non-weight-bearing in a synthetic cast with the ankle in 30° of equinus for six weeks, without intermediate cast change. Strict non-weight-bearing was chosen to avoid any risk of fall or uncontrolled loading of the construct, as this was the revision of a failed repair in a complex case, with no satisfactory secondary salvage option. Thromboprophylaxis with subcutaneous enoxaparin 4000 IU (40 mg) once daily was prescribed for 45 days to cover the period of cast immobilization and the resumption of weight-bearing. Physiotherapy was initiated at two weeks and was limited to isometric strengthening of the knee and hip during cast immobilization. Adherence, monitored by the club medical staff, was complete; the cast was never removed before six weeks. The early postoperative course was uneventful, with no wound complications and excellent scar healing (Figure 3). At six weeks, the synthetic cast was removed and a walking boot with heel wedges, progressively removed over the following three weeks, was used while progressing from partial to full weight-bearing. Concentric strengthening of the triceps surae was performed from 6 to 10 weeks, followed by eccentric strengthening from 10 to 12 weeks. At two months, the patient was walking independently without crutches with a heel-to-toe gait (Video S1). Bilateral heel rises were achieved at 15 weeks and single-leg heel rises from 17 weeks. Postoperative radiographs confirmed satisfactory positioning of the anchors and tendon transfer construct (Figure 4).

### 2.5. Follow-Up and Outcomes

**Figure 5:**
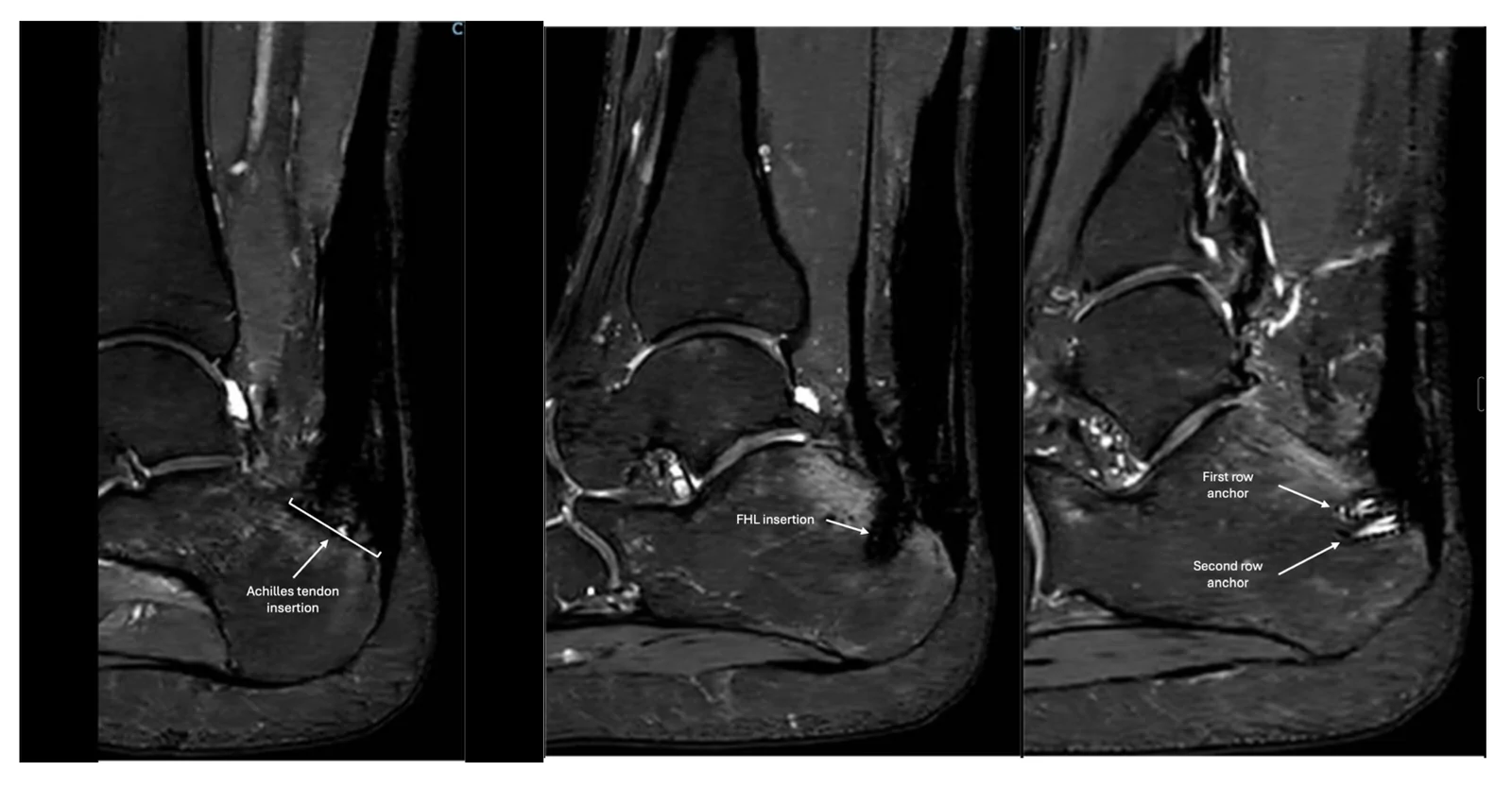
Three-month postoperative MRI—Sagittal T2-weighted fat-suppressed (T2 Fat-Sat) images showing (**left**) continuity of the reinserted Achilles tendon on the calcaneal footprint (bracket), (**middle**) the FHL tendon in its calcaneal tunnel, and (**right**) the proximal-row (**first row**) and distal-row (**second row**) anchors, without re-rupture or detachment.

Functional rehabilitation progressed without complications. At three months, MRI (radiologist’s report) showed continuity of the reinserted Achilles tendon without tear, re-rupture or detachment, with persistent thickening and low signal of the distal tendon described as residual distal tendinopathy; persistent postoperative calcaneal bone marrow edema without fracture, located anterior to the proximal-row anchors; no retrocalcaneal effusion; a calcaneal reinsertion of the FHL without complication; and normally positioned anchors (Figure 5). Running was reintroduced on an anti-gravity treadmill from four months postoperatively, with video analysis and correction of running mechanics, following consensus-based criteria for return to running after Achilles tendon repair. High-intensity running on grass was achieved by six months. Mild discomfort at the distal anchor site (visual analogue scale 2/10), occurring only during running on the track, was evaluated clinically without imaging and managed by load adjustment and modification of the shoe heel counter; it had completely resolved by 10 months postoperatively. Return to play was authorized at 8.2 months, when the Ankle-GO™ score reached 16/25, the threshold used by our team. The patient returned to official competition at 9.4 months (reserve team, 64 min), to first-team competition in the French fourth division at 9.6 months, to the first-team starting line-up at 10.3 months, and played a full 80 min match at 10.7 months. He appeared on first-team match sheets until the end of the season, including a play-off quarter-final at 12.8 months, and was still competing in the same division approximately 17 months after surgery. These competition data were obtained from publicly available match records, which may not be exhaustive. At 15 months postoperatively, the Achilles Tendon Total Rupture Score (ATRS) was 84 and the American Orthopaedic Foot and Ankle Society (AOFAS) score, which combines patient-reported and clinician-assessed items, was 90. No isokinetic or dynamometric strength testing and no assessment of hallux flexion strength were performed. The clinical and rehabilitation courses are summarized in Table 1. No surgical, thromboembolic, or other adverse or unanticipated event occurred during follow-up.

### 2.6. Patient Perspective

The patient reported that he coped very well with the surgery. For him, the most difficult period was the initial six weeks of cast immobilization without weight-bearing. He returned to the field without any particular apprehension and, at the latest follow-up, felt that he had recovered the same level as before the re-rupture.

## 3. Discussion

This case highlights a minimally invasive single-stage revision strategy for acute insertional Achilles tendon re-rupture in a semi-professional rugby player, combining endoscopic calcaneal preparation, double-row SpeedBridge™ reinsertion and flexor hallucis longus (FHL) transfer. The main learning point is that, in high-demand athletes with compromised insertional tissue, this combined approach may simultaneously address mechanical, biological, and soft-tissue challenges that often limit revision repair at the tendon–bone junction. By restoring a broad tendon footprint while augmenting the repair with a vascularized, contractile FHL tendon, the construct may enhance tendon healing and plantarflexion function without the need to rely solely on compromised residual Achilles tendon [4,13,14].

Insertional Achilles tendon ruptures and failed insertional repairs are particularly challenging in high-demand athletes. The distal Achilles insertion is a relatively hypovascular region [15], and pre-existing insertional tendinopathy, intratendinous calcification, tendon degeneration, or previous surgery leave the residual tendon retracted, deficient, and of poor quality, limiting the amount of healthy tissue available for durable tendon-to-bone repair [2,4]. Sleeve-type avulsion injuries represent a distinct and technically demanding injury pattern, often because there is insufficient residual tendon or bony fragment to allow conventional fixation [16,17]. Previous surgery may further compromise the soft-tissue envelope and local biology, increasing the risk of wound complications, infection, and re-rupture. In a competitive athlete, in whom RTS depends on near-complete restoration of plantarflexion strength and endurance, isolated repair may be mechanically and biologically insufficient.

The rationale for double-row SpeedBridge™ reinsertion is based on the need for biomechanically strong tendon–to–bone fixation. This construct restores a broad Achilles footprint and distributes the contact pressure over a larger contact area [5], which may support tendon–to–bone healing and provide enough stability for progressive rehabilitation and faster functional recovery [18,19]. Although this remains controversial, biomechanical studies suggest that the double-row technique may also have a higher peak load to failure than the single-row technique [18]. It should be noted that these biomechanical and clinical data derive from insertional tendinopathy models and have not been validated specifically for acute insertional rupture or revision surgery.

In the present case, FHL transfer was performed to provide both structural and biological augmentation of the Achilles reinsertion. The FHL is the second strongest plantarflexor of the ankle, has a similar axis of motion and activates at the same time as the *triceps surae* during gait cycle plantarflexion [20,21]. The close anatomical relationship between both tendons allows endoscopic harvesting with limited neurovascular risk. Furthermore, the distal FHL muscle belly may also improve local vascularity to the relatively hypovascular distal Achilles region, potentially supporting tendon healing. In addition, tendon hypertrophy after transfer has been reported, suggesting functional integration into active plantarflexion [22]. Although loss of hallux plantarflexion strength is a potential concern, previous studies suggest that FHL is generally well tolerated and this deficit is generally not clinically relevant, especially when distal FHL-FDL interconnections are preserved [20]. However, most of these data derive from chronic ruptures, insertional tendinopathy and primary acute ruptures, and none specifically addresses the revision of an acute insertional re-rupture.

A minimally invasive approach may be particularly useful in revision surgery. It avoids a large posterior incision over an already compromised soft-tissue envelope and preserves the surrounding soft tissues and paratenon. This may reduce the risk of wound infection, painful scarring, and adhesions, which are important disadvantages of open insertional Achilles surgery [7]; however, this evidence derives from the surgical treatment of insertional tendinopathy, and these advantages remain hypothetical in the revision setting. Posterior ankle arthroscopy also provides enhanced visualization of the calcaneal tuberosity and insertional footprint, allowing debridement, calcaneoplasty and accurate anchor placement [6,12]. Although direct comparative evidence for endoscopic revision of insertional Achilles rupture remains limited, the combination of reduced surgical morbidity and improved visualization provides a coherent rationale for this approach in selected cases.

Compared with traditional open revision strategies [23], which may involve extensive exposure, V-Y advancement, turndown flaps, or open tendon transfer, the present approach aims to provide stable fixation and biological augmentation while limiting the soft-tissue dissection associated with wound complications. Published reports of isolated endoscopic SpeedBridge™ reinsertion and endoscopic FHL transfer have described favorable outcomes and low complication rates [7,8,13]. However, these studies are generally small, non-randomized, and not specific to revision insertional rupture in elite athletes. Therefore, the findings of the present case should be interpreted cautiously. A single uncomplicated case cannot establish comparative safety or efficacy.

This strategy may be particularly relevant in competitive rugby [24]. Rugby exposes the Achilles tendon to repeated high loads during sprinting, acceleration/deceleration, jumping, cutting and contact events such as scrums and rucks [1]. These activities require a repair construct capable of tolerating both explosive concentric push-off and high eccentric braking forces. A combined construct that restores a broad bone-anchored footprint while adding a contractile biological augment may therefore be conceptually well suited to the demands of high-impact sports, where isolated repair of compromised insertional tissue may be inadequate. In the present case, the high body mass of the patient (body mass index 36.7 kg/m^2^), typical of a front-row forward, further increased the mechanical demand on the repair.

Despite its potential advantages, several limitations and risks should be acknowledged. First, the procedure is technically demanding and requires advanced arthroscopic expertise, with both endoscopic tendon reinsertion and FHL harvest. Accurate anchor placement is essential, particularly in revision bone. Arthroscopy portals-related risks include injury to the sural nerve and to the medial neurovascular structures during FHL harvest [13,25]. In the present case, no specific dissection of the sural nerve was performed, and no sensory disturbance or other complication occurred. The FHL transfer was fixed with an interference screw alone. Biomechanical studies have reported comparable primary fixation strength between interference-screw and transosseous fixation of the FHL [26,27], whereas the addition of a cortical button increased the ultimate load to failure without changing stiffness or cyclic displacement [28]. A supplementary cortical button was not used here, because the FHL served as an augmentation of a double-row Achilles reinsertion and the construct was protected by six weeks of non-weight-bearing cast immobilization; it may nevertheless be considered in heavy athletes or when bone quality is compromised. Most importantly, this report describes a single case. Patient-reported outcomes were only available at fifteen months postoperatively, limiting direct comparison. Plantarflexion strength (isokinetic or dynamometric testing) and hallux flexion strength were not objectively measured, and the Ankle-GO™ score was the only objective functional assessment. Return-to-competition data were obtained from publicly available match records, which may not be exhaustive. Therefore, the follow-up duration is insufficient to confirm durability and re-rupture risk, and no conclusions can be drawn regarding efficacy, reproducibility, or generalizability to other athletes, activity levels, or rupture patterns.

Return to sport after revision surgery for A-IATR should be individualized. Rehabilitation must balance protection of the tendon–to–bone construct and FHL transfer with the athlete’s need for progressive loading, strength restoration, and sport-specific conditioning. Progression should be guided by objective measures of strength, range of motion, functional performance, and psychological readiness rather than by time-based guidelines [2,29]. Although reported return-to-play outcomes after endoscopic FHL transfer in professional athletes are encouraging, specific criteria and protocols are still missing [2,29]. In the present case, return to running followed recently published consensus criteria [30], and return to play was authorized on the basis of the Ankle-GO™ score, a composite score validated after Achilles tendon repair [31]. The threshold of 16 points used by our team has not itself been validated as a return-to-play cut-off.

## 4. Conclusions

In this semi-professional rugby player with an acute insertional Achilles tendon re-rupture, a single stage combination of endoscopic calcaneal SpeedBridge^TM^ reinsertion and flexor hallucis longus transfer was followed by an uncomplicated course, a return to official competition at 9.4 months and sustained participation at approximately 17 months. This all-endoscopic approach combines double-row reinsertion and biological augmentation, aiming to restore the Achilles tendon footprint while minimizing soft-tissue morbidity. This case may be of particular interest to foot-and-ankle and sports surgeons and suggests a potential role of minimally invasive Achilles reinsertion techniques in high-level athletic rehabilitation.

## Figures and Tables

**Table 1:** Clinical and rehabilitation courses.

Timeline	Assessment	Findings/Milestones
Day 0	Surgery	Endoscopic double-row SpeedBridge™ reinsertion combined with FHL tendon transfer. Non-weight-bearing in a synthetic cast (30° equinus) initiated. Thromboprophylaxis (enoxaparin 40 mg once daily) prescribed for 45 days.
2 weeks	Rehabilitation	Physiotherapy initiated (isometric knee and hip exercises). Uneventful wound healing with no postoperative complications.
6 weeks	Clinical + Imaging	Synthetic cast removed; walking boot with heel wedges removed over 3 weeks. Progression from partial to full weight-bearing. Concentric strengthening (6–10 weeks), then eccentric strengthening (10–12 weeks). Radiographs confirmed satisfactory positioning of the anchors and tendon transfer construct.
2 months	Clinical	Independent walking without crutches with heel-to-toe gait.
3 months	Imaging	Control MRI: continuity of the reinserted tendon without re-rupture or detachment; persistent distal thickening (residual tendinopathy).
4 months	Rehabilitation	Running reintroduced on an anti-gravity treadmill with gait-video analysis; bilateral (15 weeks) then single-leg (17 weeks) heel rises achieved.
6 months	Clinical	High-intensity running on grass; mild distal anchor-site discomfort during track running (VAS 2/10).
8 months	Return-to-play clearance	Ankle-GO™ 16/25 at 8.2 months; return to play authorized.
9.4 months	Return to competition	First official match (reserve team, 64 min); first-team match (French fourth division) at 9.6 months; first start at 10.3 months; full 80 min match at 10.7 months.
10 months	Clinical	Complete resolution of distal anchor-site discomfort.
15 months	Clinical	No surgical, thromboembolic or other adverse events during follow-up. ATRS 84, AOFAS 90.
17 months	Follow-up	Still competing in the French fourth division.

AOFAS, American Orthopaedic Foot and Ankle Society score; ATRS, Achilles Tendon Total Rupture Score; FHL, flexor hallucis longus; MRI, magnetic resonance imaging; VAS, visual analogue scale.

## Data Availability

The original contributions presented in this study are included in the article/Supplementary Material. Further inquiries can be directed to the corresponding author.

## Supplementary Materials

The following supporting information can be downloaded at https://www.mdpi.com/article/10.3390/jcm15197725/s1, Video S1: Thompson test and independent heel-to-toe walking at two months postoperatively.

## Abbreviations

The following abbreviations are used in this manuscript:

A-IATR	Acute insertional Achilles tendon rupture
AOFAS	American Orthopaedic Foot and Ankle Society Score
ATRS	Achilles Tendon Total Rupture Score
FHL	Flexor hallucis longus
MRI	Magnetic resonance imaging
RTS	Return to sport
VAS	Visual analogue scale
